# Investigations in West Syndrome: Which, When and Why

**DOI:** 10.15844/pedneurbriefs-29-6-1

**Published:** 2015-06

**Authors:** Richard E. Appleton

**Affiliations:** 1 The Roald Dahl EEG Unit, Paediatric Neurosciences Foundation, Alder Hey Children's Hospital, Liverpool, UK

**Keywords:** Infantile Spasms, West syndrome, Investigations

## Abstract

Investigators from the National Infantile Spasms Consortium (NISC) in the USA studied the etiology of new-onset infantile spasms (IS) in 251 infants (mean age at onset, 7.1, range, 0.1-22.7 months).

Investigators from the National Infantile Spasms Consortium (NISC) in the USA studied the etiology of new-onset infantile spasms (IS) in 251 infants (mean age at onset, 7.1, range, 0.1-22.7 months). The study aim was to evaluate the yield of genetic and/or metabolic investigations in the identification of an etiology of IS within three months of diagnosis when no cause had been found after an initial clinical assessment and brain MRI. Clinical evaluation and brain MRI identified a specific etiology in 138 of 250 (55%) children. Additional etiologies were identified in another 23 cases following genetic (18) and “metabolic” (5) studies. No correlation was found between type of health insurance and the genetic and metabolic testing. The NISC proposed a cost-effective workup for those children in whom initial clinical assessment and brain MRI failed to identify a cause: array comparative genomic hybridization followed by an epilepsy gene panel, and, if genetic testing is not “definitive”, metabolic studies. [1]

COMMENTARY: West syndrome (WS) remains one of the most iconic but also enigmatic of the ‘epileptic encephalopathies’. The focus over the past 40 years has been on treatment, and prognosis – for both the epilepsy and developmental (cognitive) outcome. Debate continues as to which is(are) the most important prognostic factor(s), particularly for developmental outcome. The principal ones are the time to diagnosis (and treatment), and the underlying cause. Most studies evaluating outcome (spasm-suppression and development) by time to diagnosis or by specific treatments have largely failed to correct by the underlying cause [2, 3, 4], other than in children with tuberous sclerosis complex [5] or Down syndrome [6]. Wirrell *et al*. take an interesting and refreshing approach to etiology. Overall, the results are predictable, at least to clinicians that have been managing children with WS for many years. Undoubtedly, more of the iceberg-tip of ‘cryptogenic’ WS will melt with advances in molecular genetics and possibly more routine higher-resolution (e.g. 5T MRI) neuroimaging. It is also likely that genetic results will be more rapidly available than the three-month census used in this study [1]. Consequently, this should improve counselling and may obviate the need for additional and potentially more invasive investigations as has been shown in Dravet syndrome [7].

The issue of the cost-effectiveness of investigations in WS is important. The cumulative cost of all potential investigations is significant, particularly for those families with no or only limited health insurance, as in the US. Despite the NHS in the UK, there is a financial impact of repeat neuro-imaging and particularly next generation sequencing (epilepsy gene panel), which though small is a contributing factor in the spiraling costs of the NHS. This will be even more relevant to developing countries.
